# Assessment of the peripheral microcirculation in patients with and without shock: a pilot study on different methods

**DOI:** 10.1007/s10877-019-00423-8

**Published:** 2019-11-21

**Authors:** Roberto Rabello Filho, Renato Carneiro de Freitas Chaves, Murillo Santucci Cesar Assunção, Ary Serpa Neto, Flavia Manfredi De Freitas, Maria Laura Romagnoli, Eliézer Silva, Bernardo Lattanzio, Arnaldo Dubin, Thiago Domingos Corrêa

**Affiliations:** 1 Departamento de Terapia Intensiva, Hospital Israelita Albert Einstein, Av. Albert Einstein, 627/701, 5th Floor, São Paulo, 05651-901 Brazil; 2 Departamento de Anestesiologia, Irmandade da Santa Casa de Misericórdia de Santos, Santos, Brazil; 3 Department of Intensive Care, Academic Medical Center, University of Amsterdam, Amsterdam, The Netherlands; 4 Cátedra de Farmacología Aplicada, Facultad de Ciencias Médicas, Universidad Nacional de La Plata, La Plata, Argentina; 5 Servicio de Terapia Intensiva, Sanatorio Otamendi, Buenos Aires, Argentina

**Keywords:** Critical care, Shock, Hemodynamics, Microcirculation, Oxygen consumption, Near-infrared spectroscopy

## Abstract

Microvascular dysfunction has been associated with adverse outcomes in critically ill patients, and the current concept of hemodynamic incoherence has gained attention. Our objective was to perform a comprehensive analysis of microcirculatory perfusion parameters and to investigate the best variables that could discriminate patients with and without circulatory shock during early intensive care unit (ICU) admission. This prospective observational study comprised a sample of 40 adult patients with and without circulatory shock (n = 20, each) admitted to the ICU within 24 h. Peripheral clinical [capillary refill time (CRT), peripheral perfusion index (PPI), skin-temperature gradient (Tskin-diff)] and laboratory [arterial lactate and base excess (BE)] perfusion parameters, in addition to near-infrared spectroscopy (NIRS)-derived variables were simultaneously assessed. While lactate, BE, CRT, PPI and Tskin-diff did not differ significantly between the groups, shock patients had lower baseline tissue oxygen saturation (StO_2_) [81 (76–83) % vs. 86 (76–90) %, p = 0.044], lower StO_2_min [50 (47–57) % vs. 55 (53–65)  %, p = 0.038] and lower StO_2_max [87 (80–92) % vs. 93 (90–95) %, p = 0.017] than patients without shock. Additionally, dynamic NIRS variables [recovery time (r = 0.56, p = 0.010), descending slope (r = − 0.44, p = 0.05) and ascending slope (r = − 0.54, p = 0.014)] and not static variable [baseline StO_2_ (r = − 0.24, p = 0.28)] exhibited a significant correlation with the administered dose of norepinephrine. In our study with critically ill patients assessed within the first twenty-four hours of ICU admission, among the perfusion parameters, only NIRS-derived parameters could discriminate patients with and without shock.

**Electronic supplementary material:**

The online version of this article (10.1007/s10877-019-00423-8) contains supplementary material, which is available to authorized users.

## Introduction

Early and proper resuscitation is essential to restore tissue perfusion and to preserve cell function in circulatory shock [1]. Although international guidelines recommend targeting macro-hemodynamic parameters such as mean arterial blood pressure (MAP), central venous pressure (CVP), central venous oxygen saturation (ScvO_2_) and blood lactate levels during resuscitation [2], several clinical studies failed to demonstrate a clear relationship between macro and micro-hemodynamics, which has been termed as “hemodynamic incoherence” [3, 4].

Microcirculation is a heterogeneous, dynamic and autonomous system with complex regulation and homeostasis [5]. Several authors have demonstrated that derangements in microcirculation are related to multiple organ failure and death in critically ill patients [6, 7]. For instance, it has been shown in septic patients that microvascular perfusion improves faster in survivors than in non-survivors [8]. More interestingly, even after reestablishing systemic hemodynamics, microcirculation parameters may remain impaired while severity of microvascular dysfunction is also related to poor clinical outcomes [8, 9].

Currently, the diagnosis of shock is based on systemic arterial hypotension, hyperlactatemia and clinical signs of tissue hypoperfusion, which may be apparent at the bedside in three ways: cutaneous (cold and clammy skin), renal (decreased urine output) and neurologic (altered mental state) [10]. Unlike renal or neurologic dysfunction, skin abnormalities may be subjective. Relevant cutaneous markers of tissue perfusion such as capillary refill time (CRT), peripheral perfusion index (PPI), skin-temperature gradient (Tskin-diff), in addition to tissue oxygen saturation (StO_2_) are not cited in the definition of circulatory shock in large international studies or in the consensus, and the assessment of most of these quantitative peripheral perfusion parameters has not been incorporated into routine clinical practice so far [11, 12].

Moreover, considering the dissociation between macro- and microcirculatory compartments, the assessment of tissue perfusion in intensive care unit (ICU) patients is of paramount importance [13]. Despite technological advances in this field, the direct identification of severe microcirculatory alterations remains difficult at bedside. Several controversies remain about the behavior of cutaneous peripheral perfusion parameters according to the severity of shock [14, 15]. For instance, there is considerable overlap between pathological values and the StO_2_ values obtained under physiological conditions [16]. Clinicians should rely on a combination of parameters in detecting “occult” shock and a simultaneous analysis of clinical and laboratory tissue perfusion parameters, in addition to NIRS static and dynamic-derived variables could provide relevant information. Therefore, the objective of this exploratory study was to perform a comprehensive, quantitative and noninvasive evaluation of peripheral perfusion and to investigate the microcirculatory parameters that discriminate patients with and without circulatory shock.

## Materials and methods

### Study design and setting

This prospective single-center observational study was conducted in a 37 bed, open medical-surgical ICU of a tertiary care hospital in São Paulo, Brazil. This study was approved by the institutional review board, and written informed consent was obtained from each study participant or their next of kin.

### Participants

Adult (≥ 18 years old) patients with and without circulatory shock within 24 h of ICU admission were eligible for inclusion. Moribund, palliative care and pregnant patients were excluded.

Circulatory shock was defined as hypotension [systolic blood pressure (SBP) < 100 mm Hg or MAP < 70 mm Hg] or the need of vasopressors (norepinephrine ≥ 0.1 mcg/kg/min or epinephrine ≥ 0.1 mcg/kg/min for at least 1 h) despite adequate fluid resuscitation and the presence of at least one sign of tissue hypoperfusion, such as increased lactate levels (> 2 mmol/L), mottled skin, altered mental status or urinary output < 0.5 mL/Kg/h [11].

### Measurements

Age, gender, reason for ICU admission, comorbidities and simplified acute physiology (SAPS) III score [17] were recorded at ICU admission. The use of vasopressors (norepinephrine and epinephrine), inotropes, corticosteroids and the need of renal replacement therapy (RRT) were recorded at the time of study inclusion. Systemic hemodynamic variables, ventilatory parameters and the administered dose of norepinephrine were recorded simultaneously with the evaluation of the peripheral perfusion parameters. Arterial blood gas analyses were recorded from the closer time of inclusion in the study. Urine output and fluid balance were recorded from the ICU admission until study inclusion. Sequential organ failure assessment (SOFA) score [18] was recorded over the first 24 h following ICU admission. Finally, ICU, hospital and 28-day mortality were recorded.

All patients were monitored using a multi-parameter monitor, and global hemodynamic variables including heart rate, CVP and MAP were obtained by using standard equipment. Cardiac output was measured in shock patients with continuous pulse contour cardiac analysis (FloTrac/EV1000 clinical platform; Edwards Lifesciences LLC, Irvine, CA, USA).

#### Peripheral perfusion parameters

CRT was measured by applying pressure on the distal phalanx of the index finger for 15 s [19]. A chronometer recorded the time until return to normal color and a value < 5 s was defined as the limit of normality [19].

PPI is a non-invasive method derived from the photoelectric signal of the pulse oximeter (Masimo^®^ SET Radical-7, Masimo Corporation, Irvine, CA, USA), which displays a range from 0.02% (very low pulse strength) to 20.0% (very high pulse strength) [20]. The PPI reflects changes in peripheral circulation and a value < 1.4 defines the presence of poor peripheral perfusion (peripheral vasoconstriction) [21].

Finally, Tskin-diff is a traditional index for identifying peripheral vasoconstriction [21]. We measured Tskin-diff with two skin probes (Hewlett-Packard 21078A; Hewlett-Packard, Palo Alto, CA, USA) placed on the index finger and on the radial side of the forearm, midway between the elbow and the wrist [22]. A threshold of Tskin-diff > 2 °C was adopted to define vasoconstriction [23].

#### NIRS monitoring and analysis

Thenar StO_2_ was continuously monitored using the InSpectra StO_2_ Tissue Oxygenation Monitor (model 650; Hutchinson Technology, Hutchinson, MN, USA) with a 15-mm probe over the thenar eminence [15]. After 3 min of minimal variation of StO_2_ (NIRS signal stabilization), the basal StO_2_ was recorded [15]. The vascular occlusion test (VOT) was performed using a conventional sphygmomanometer pneumatic cuff [15]. VOT starts with inflation of the cuff to 30 mm Hg above SBP for 3 min [15]. Upon completion of the ischemic period (3 min), the occluding cuff was rapidly deflated to 0 mm Hg, and StO_2_ was continuously recorded during the reperfusion phase for 5 min [15].

Thenar StO_2_ represents the local balance between O_2_ delivery and O_2_ consumption. Dynamic changes in StO_2_ during a brief episode of ischemia enable analysis of microvascular dysfunction [15, 24]. The descending slope is a reflection of local oxygen consumption, providing an index of O_2_ extraction during the transient interruption of arterial inflow [25]. Hypoxia induces dilation of precapillary arterioles, favoring opening of the microcirculation. Thus, the ascending slope represents the early reperfusion related to increase of arterial inflow immediately after the end of VOT [24]. Reactive hyperemia is a vasoreactivity test related to microvascular reserve in previously patent capillaries and recruiting additional capillaries [24]. The area under the curve of reactive hyperemia evaluates the oxygen extraction capacity, reflecting the degree of hyperemic reaction, when the vascular tone is decreased [24].

Research software (Hutchinson Technology Inc., Hutchinson, MN, USA) was used to analyze NIRS-derived parameters. StO_2_ (%) and tissue hemoglobin index (THI) were measured at baseline [15]. The descending slope (%/minute) was calculated from the StO_2_ baseline until the minimum value of StO_2_ (StO_2_min) immediately after the end of VOT [15], while the ascending slope (%/minute) was calculated from the StO_2_min immediately after the end of the VOT until the maximum value of StO_2_ (StO_2_max) [15]. The area under the curve of reactive hyperemia was calculated from the StO_2_max until StO_2_ returns to baseline [15].

### Statistical analysis

A convenience sample of 40 patients with and without circulatory shock (n = 20, each) was established. Categorical variables were expressed as absolute and relative frequencies, and continuous variables were expressed as median (25th–75th ‰). Normality was addressed with the Kolmogorov–Smirnov test. Proportions between groups (patients with and without circulatory shock) were compared with Chi square test or Fisher’s exact test. Continuous variables were compared between groups with independent samples *t* test or Mann–Whitney *U*-test in case of non-normal distribution. Correlation between peripheral perfusion parameters, NIRS**-**derived parameters (independent variables) and SOFA score (dependent variable) were assessed in the whole cohort with Pearson correlation coefficient. Correlation between dose of norepinephrine (independent variable) and NIRS-derived parameters (dependent variable) was also assessed with Spearman´s correlation coefficient.

All analyses were performed using IBM SPSS (version 23.0) and GraphPad Prism software version 7.02 (Graphpad Software, Inc., La Jolla, CA, USA). A p value of less than 0.05 was considered statistically significant.

## Results

### Patients' characteristics

Patients with shock (80% septic shock; 20% cardiogenic shock) were older [66 (56–73) vs. 50 (44–60) years, p = 0.038] and had higher SOFA [(8 (6–10) vs. 4 (1–5), p < 0,001] and SAPS III [53 (45–65) vs. 30 (22–46), p < 0,001] scores than patients without shock (Table 1). Medical patients were the majority of patients with circulatory shock (60%), while 70% of patients without shock were surgical (p = 0.057) (Table 1). Patients with shock received more vasopressors [20 (100%) vs. 4 (20.0%), p < 0.001], mechanical ventilation [10 (50%) vs. 1 (5%), p = 0.003] and intravenous corticosteroid [6 (30%) vs. 0 (0%), p = 0.020] compared with patients without shock (Table 1).

**Table 1:** Characteristics of critically ill patients

Characteristics	Shock Patients (n = 20)	Non-shock Patients (n = 20)	P value
Age, years	66 (56–73)	50 (44–60)	0.038^a^
Men, n (%)	9 (45.0)	14 (70.0)	0.200^b^
SAPS III score	53 (45–65)	30 (22–46)	<0.001^c^
SOFA score	8 (6–10)	4 (1–5)	<0.001^c^
Time between ICU admission and study inclusion, h	15 (11–19)	14 (8–18)	0.225^c^
Type of admission, n (%)			0.057^b^
Medical	12 (60.0)	6 (30.0)	
Surgical	8 (40.0)	14 (70.0)	
Admission source, n (%)			0.154^b^
Emergency department	9 (45.0)	4 (20.0)	
Operating room	7 (35.0)	14 (70.0)	
Step down unit	1 (5.0)	1 (5.0)	
Other ICU	3 (15.0)	1 (5.0)	
Underlying disease, n (%)			
Systemic hypertension	7 (35.0)	8 (40.0)	0.744^b^
Diabetes mellitus	5 (25.0)	3 (15.0)	0.695^d^
Coronary insufficiency	3 (15.0)	4 (20.0)	1.000^d^
Congestive heart failure	3 (15.0)	1 (5.0)	0.605^d^
Transplantation	1 (5.0)	1 (5.0)	1.000^d^
Non-operative admission diagnoses, n (%)			
Sepsis	7 (35.0)	2 (10.0)	
Cardiovascular	2 (10.0)	1 (5.0)	
Respiratory	3 (15.0)	0 (0.0)	
Gastrointestinal	0 (0.0)	2 (10.0)	
Metabolic	0 (0.0)	0 (0.0)	
Trauma	0 (0.0)	0 (0.0)	
Hematologic	0 (0.0)	1 (5.0)	
Operative admission diagnoses, n (%)			
Cardiovascular	6 (30.0)	2 (10.0)	
Gastrointestinal	2 (10.0)	1 (5.0)	
Renal	0 (0.0)	5 (25.0)	
Neurologic	0 (0.0)	2 (10.0)	
Others	0 (0.0)	4 (20.0)	
Intravenous fluids administered*, L	1850 (1000–3225)	1000 (1000–2500)	0.552^a^
Vasoactive drugs, n (%)	20 (100.0)	4 (20.0)	<0.001^b^
Norepinephrine, n (%)	20 (100.0)	1 (5.0)	<0.001^b^
μg/kg/min	0.16 (0.10–0.41)	0.013 (0.13–0.13)	0.095^a^
Dobutamine, n (%)	8 (40.0)	2 (10.0)	0.065^d^
μg/kg/min	4.0 (2.8–7.4)	3.5 (2.0–5.0)	0.533^a^
Epinephrine, n (%)	2 (10.0)	0 (0.0)	0.487^d^
μg/kg/min	0.13 (0.13–0.13)		
Mechanical ventilation, n (%)	10 (50.0)	1 (5.0)	0.003^d^
Renal replacement therapy, n (%)	0 (0.0)	1 (5.0)	1.000^d^
Intravenous corticosteroid, n (%)	6 (30.0)	0 (0.0)	0.020^d^

Values represent median (IQR) or n (%). * Intravenous fluids include crystalloids and colloids administered from ICU admission until study inclusion

P values were calculated with (a) Mann–Whitney *U* test, (b) Chi square test, (c) Independent *t*-test, (d) Fisher exact test

### Systemic hemodynamics and peripheral perfusion parameters

**Fig. 1:**
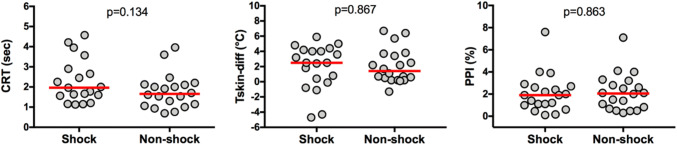
Peripheral perfusion parameters. *CRT* capillary refill time, *Tskin-diff* forearm-to-fingertip skin temperature gradient, *PPI* peripheral perfusion index. Red horizontal bars represent median

Systemic hemodynamics and arterial blood gas results are presented in Table 2. Shock patients presented significant higher heart rate (HR) [95 (79–105) bpm vs. 81 (70–93) bpm; p = 0.024] and lower MAP [70 (66–73) mm Hg vs. 81 (71–100) mmHg; p = 0.002] compared to non-shock patients (Table 2). Peripheral perfusion parameters (CRT, Tskin-diff and PPI) (Fig. 1) and arterial blood gas results (Table 2) did not differ between groups.

**Table 2:** Baseline systemic hemodynamics and arterial blood gas analysis

Characteristics	Shock Patients (n = 20)	Non-shock Patients (n = 20)	P value
Heart rate (bpm)	95 (79–105)	81 (70–93)	0.024^a^
MAP (mmHg)	70 (66–73)	81 (71–100)	0.002^a^
Cardiac index (L/min/m^2^)	3.1 (1.9–5.2)		
Arterial lactate (mmol/L)	3.2 (1.9–4.5)	2.4 (0.8–2.9)	0.095^b^
ScvO_2_ (%)	75 (54–83)		
Arterial (pH)	7.35 (7.33–7.39)	7.38 (7.34–7.41)	0.354^a^
PaO_2_ (mmHg)	130 (98–142)	97 (74–117)	0.134^b^
PaCO_2_ (mmHg)	33.6 (28.4–38.6)	36.8 (35.7–41.8)	0.304^b^
Base excess (mEq/L)	− 5.8 (− 8.4 to − 3.1)	− 3.5 (− 3.8 to − 0.7)	0.248^b^

Values represent median (IQR)

*MAP* mean arterial blood pressure, *ScvO*_*2*_ central venous oxygen saturation, *PaO*_*2*_ partial pressure of arterial oxygen, *PaCO*_*2*_ partial pressure of arterial carbon dioxide

P values were calculated with the use of (a) Mann–Whitney *U* test and (b) Independent *t*-test. * Systemic hemodynamic variables were recorded at the time of study inclusion, simultaneously with the evaluation of the peripheral perfusion parameters

^#^Arterial blood gas analyses were recorded from the closer time of inclusion in the study

### NIRS-derived parameters

**Fig. 2:**
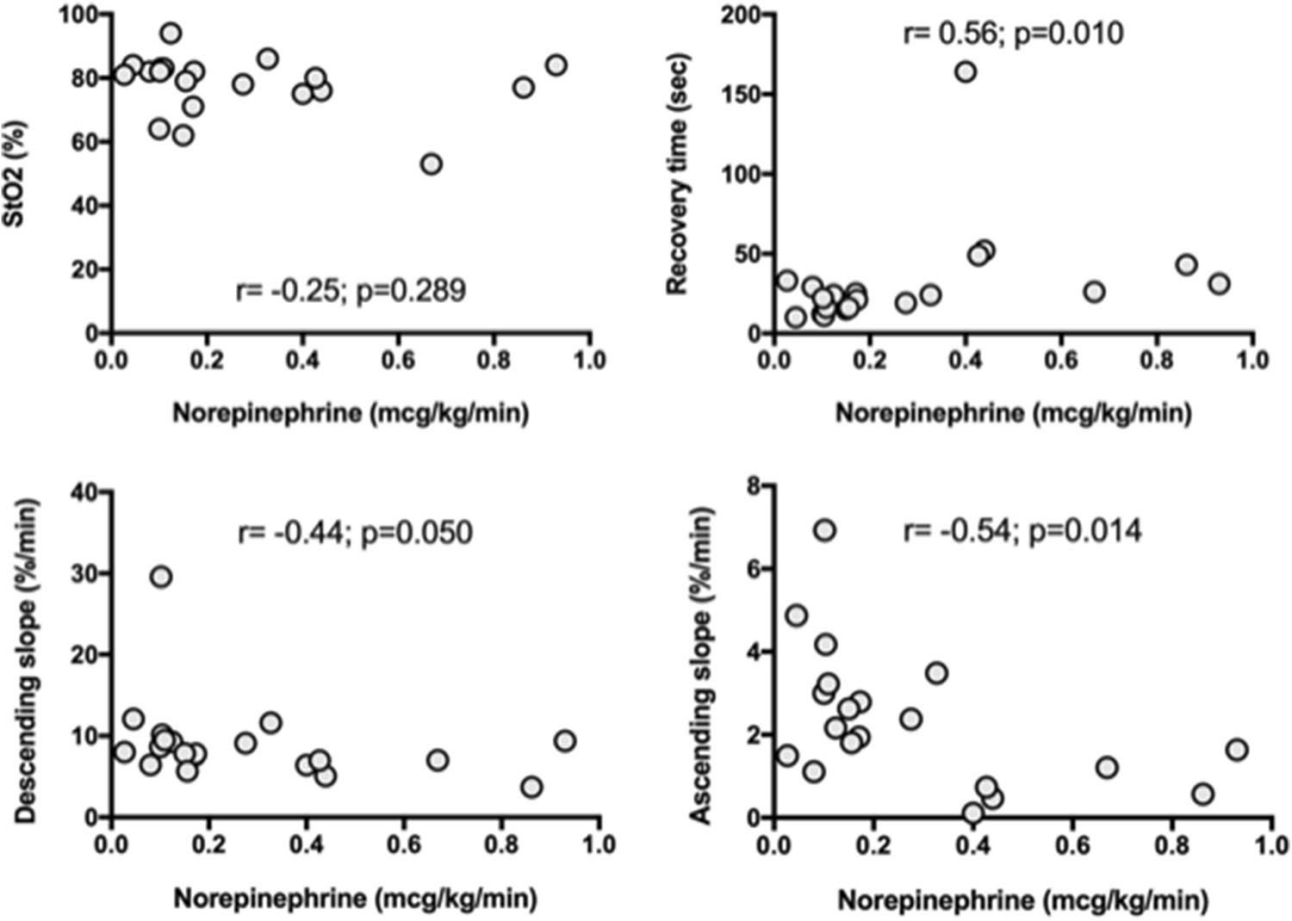
Correlation between dose of norepinephrine and NIRS-derived parameters. *StO*_*2*_ basal tissue oxygen saturation

Shock patients had significant lower THI [11.3 (9.6–14.1) vs. 13.7 (10.5–15.0), p = 0.039], baseline StO_2_ [81 (76–83) % vs. 86 (76–90) %, p = 0.044], StO_2_min [50% (47–57) % vs. 55% (53–65) %, p = 0.038] and StO_2_max [87 (80–92) % vs. 93 (90–95) %, p = 0.017] than critically ill patients without shock (Table 3). Descending slope, ascending slope, recovery time, and hyperemia area did not differ between groups (Table 3). Dynamic NIRS variables [recovery time (r = 0.56, p = 0.010), descending slope (r = − 0.44, p = 0.05) and ascending slope (r = − 0.54, p = 0.014)] and not a static variable [baseline StO_2_ (r = − 0.24, p = 0.28)] exhibited a significant correlation with the administered dose of norepinephrine in patients with shock (Fig. 2). The NIRS-derived parameters were recorded with a median time of 13 (9–18) hours after ICU admission and all patients were in normothermia.

**Table 3:** Near infrared spectroscopy parameters

Characteristics	Shock Patients (n = 20)	Non- shock Patients (n = 20)	P value
THI	11.3 (9.6–14.1)	13.7 (10.5–15.0)	0.039^a^
StO_2_ (%)	81 (76–83)	86 (76–90)	0.044^a^
StO_2_ min (%)	50 (47–57)	55 (53–65)	0.038^b^
StO_2_ max (%)	87 (80–92)	93 (90–95)	0.017^a^
Descending slope (%/min)	7.9 (6.7–9.4)	8.4 (6.0–9.8)	0.965^b^
Ascending slope (%/s)	2.1 (1.2–3.1)	2.2 (1.6–3.4)	0.559^a^
Recovery time (s)	24.0 (16.0–32.0)	16.5 (13.0–24.0)	0.093^b^
StO_2_max–StO_2_min (%)	7 (5–11)	7 (4–12)	0.926^a^
Hyperemia area	8.6 (4.7–15.2)	8.9 (4.0–13.3)	1.000^b^

Values represent median (IQR)

*THI* tissue hemoglobin index, *StO*_*2*_ tissue oxygen saturation, *StO*_*2*_*min* minimum StO_2_ after arterial occlusion test, *StO*_*2*_*max* maximum StO_2_ after arterial occlusion

P values were calculated with the use of (a) independent *t* test and (b) Mann–Whitney *U* test

### Organ dysfunction, length of stay and mortality

Capillary refill time (r = 0.40, p = 0.011) but not Tskin-diff, PPI and NIRS-derived parameters exhibited a positive correlation with SOFA score (Table S1 Supplementary material). The CRT was also the only peripheral perfusion variable with a significant difference between survivors and non-survivors [1.63 (1.20–1.96) s vs. 3.01 (2.25–3.95) s, p = 0.012]. Patients with shock had a higher ICU [30% (6/20 patients) vs. 0% (0/20 patients), p = 0.020], hospital [30% (6/20 patients) vs. 0% (0/20 patients), p = 0.020] and 28-day [25% (5/20 patients) vs. 0% (0/20 patients), p = 0.047] mortality than non-shock patients.

## Discussion

We found that, differently from clinical and laboratory peripheral perfusion parameters, NIRS-derived static and dynamic parameters discriminated between shock and non-shock patients within the first 24 h of ICU admission. The dynamic parameters derived from NIRS were inversely correlated to the administered dose of norepinephrine. Additionally, although similar values in shock and non-shock patients, CRT exhibited a positive correlation with SOFA score, and was the only peripheral perfusion variable with a significant difference between survivors and non-survivors.

Microcirculatory dysfunction has been associated with increased morbidity and mortality in critically ill patients [6]. For instance, persistent abnormalities in sublingual microcirculatory, and not global hemodynamic parameters, discriminated between septic shock survivors and septic patients dying of multiple organ failure [6]. Most importantly, due to the absence of a clear relationship between macro and micro-hemodynamics (hemodynamic incoherence) [3], the achievement of systemic resuscitation goals may not translate into improved microcirculation and can contribute to fluid overload and additional exposure to catecholamines [26].

The subjective assessment of peripheral perfusion with physical examination of the skin can be a valuable adjunct in hemodynamic monitoring during circulatory shock [9]. Lima et al. demonstrated that hemodynamically stable patients have an increased risk of developing organ dysfunction if abnormal clinical signals of peripheral perfusion, such as CRT, Tskin-diff and PPI are detected [9]. Moreover, a prolonged CRT after 6 h of resuscitation has been shown to be predictive of 14-day mortality in septic shock patients [27]. Other observational studies have also demonstrated a strong relationship between skin clinical parameters and higher mortality in patients with shock, such as skin temperature gradients and mottling [28, 29]. In addition, a meta-analysis involving 20 studies and 717 septic patients showed that survivors had higher levels of StO_2_ compared with non survivors at different times of measurements [30]. In our study, we observed that only CRT exhibited a positive correlation with SOFA score and hospital mortality. We studied a mixed population of ICU patients, resuscitated before study enrollment, as demonstrated by the cardiac index (CI) and ScvO_2_ values, and without serial StO_2_ measurements overtime. Our exploratory study was not powered to investigate associations with mortality. However, the present data supports the hypothesis that NIRS measurements may be more useful when analyzed along with other peripheral perfusion variables, particularly CRT.

Recent studies have suggested that StO_2_ values can be used as a screening tool in potentially critical patients [31, 32]. Bazerbashi et al. demonstrated that patients with a static value of StO_2_ < 70% at presentation in the emergency department (ED) were associated with a 2.64 times increase in ICU admission compared to those with StO_2_ of > 70% [32]. Another prior study evidenced more severe organ dysfunction in septic patients who consistently presented StO_2_ < 70% during the first 8 h of resuscitation [32]. Furthermore, there was no significant relationship between low StO_2_ values and global hemodynamic parameters, such as HR and MAP [32].

Our findings are consistent with previous studies showing that peripheral blood flow variables may be altered in different experimental and clinical shock conditions [6, 33, 34]. In this regard, a recent study with adult patients presenting to the ED with suspected sepsis diagnosis, used a similar noninvasive optical device to measure the muscle oxygenation (MOx) and found that MOx could stratify patients in mild and moderate shock, defined by degrees of systemic hemodynamic variables and lactate levels [35]. Our study expands these previous observations demonstrating that changes in NIRS-derived variables assessed early in a mixed ICU population can detect the presence of shock.

By inducing an ischemic stress, VOT provides important information on tissue O_2_ extraction and microvascular reactivity [30, 36]. Dynamic VOT parameters had a higher accuracy in detecting microvascular dysfunction in critically ill patients than static values [30, 36]. In a mix critically ill adults’ population, Donati et al. showed that the desaturation rate tended to be slower in the late ischemic phase in patients with sepsis, hypotension, high lactate levels or with norepinephrine administration (conditions of a likely hemodynamic instability) [37]. Although our study, involving a smaller population, evidenced similar descending slope rates between shock and non-shock patients, we observed lower values of StO_2_min in patients with shock compared with patients without shock, probably due to the imbalance between supply and demand of oxygen and lower auto regulatory reserve [38].

Reactive hyperemia can evaluate the tissue’s ability to adjust oxygen extraction capabilities to oxygen delivery after a hypoxic stimulus induced by VOT [39]. The difference between the maximum StO_2_ during the hyperemic phase and baseline StO_2_ (ΔStO_2_) can be used to estimate the microcirculatory reactivity [39]. Unlike our findings, a previous study involving 72 patients with severe sepsis or septic shock showed lower slopes (ΔStO_2_) in patients with shock than non-shock patients [24]. More interestingly, there was no correlation between slope and norepinephrine dose [24]. Nevertheless, we found a moderate negative correlation between the norepinephrine administered dose and dynamic measurements derived from NIRS (recovery time, descending slope and ascending slope) in our study. Our results are consistent with other previous results suggesting that the local vasoconstriction mediated by a pharmacological intervention might be deleterious, regardless of the optimization of global hemodynamic variables [19, 40, 41]. In addition, our data may suggest that the potentially harmful effect of vasopressor administration on microcirculation may be dose dependent.

Compared with other techniques, the advantages of NIRS are its noninvasiveness, real-time continuous monitoring, with a relatively inexpensive and small device that is easy to use [35]. However, the utility of NIRS in the management of critically ill patients is still a matter of debate. A recent randomized trial study of StO_2_-guided resuscitation with sepsis or septic shock patients at ICU admission found that the inclusion of StO_2_ > 80% as a target in the algorithm for early goal-directed therapy did not improve clinical outcomes [42]. Moreover, this experimental algorithm of resuscitation was associated with more time on mechanical ventilation, more blood transfusion and more use of inotropes [42]. However, another randomized controlled pilot study was performed comparing a peripheral perfusion–guided early fluid resuscitation with a classical strategy based on MAP, CVP and CI in septic shock patients admitted to the ICU [43]. Peripheral perfusion was assessed through CRT, Tskin-diff, PPI and StO_2_ [43]. The strategy based on clinical tissue perfusion assessment demonstrated reduction in fluid therapy volume in the first 72 h, reduction in hospital length of stay and lower organ failure scores [43].

The role of the clinical assessment of peripheral perfusion as a target during early resuscitation in shock was further evaluated in a recent large-scale multicenter randomized trial comparing peripheral perfusion–targeted resuscitation to blood lactate level–targeted resuscitation during an 8-h intervention period [44]. Patients were randomized to a stepwise resuscitation protocol aimed at either normalizing CRT or decreasing lactate levels at rates greater than 20% per 2 h [44]. Peripheral perfusion–targeted resuscitation was associated with less organ dysfunction at 72 h. Despite the absence of significant differences in all-cause 28-day mortality, goal-directed therapy protocols based on serial measurements of CRT is a promising therapeutic approach [44].

This study has some limitations. First, the number of patients included in this study was limited. Moreover, correlations between perfusion parameters, SOFA score and between doses of norepinephrine were not adjusted for confounders. Therefore, the risk of spurious false-positive and false-negative findings must be considered. Second, administered treatment (e.g., dobutamine, fluids and corticosteroids) was not similar between the groups and our patient population with shock was heterogeneous, which may have affected our results. Third, peripheral tissue perfusion parameters alter in a constant dynamic manner and we included patients at variable time points in admission. Although we performed a comprehensive evaluation of several microcirculatory parameters and the assessment of peripheral perfusion could aid in the diagnosis of shock, it is not clear what the clinical consequences should be when these measurements are taken at varying time points and following variable interventions.

## Conclusions

In this prospective, single center observational study, we found that NIRS-derived static and dynamic parameters discriminated between shock and non-shock patients in the first 24 h of ICU admission. In patients with shock, the application of VOT has a potential for a more comprehensive evaluation of peripheral perfusion and dynamic NIRS-derived variables may be associated with norepinephrine dose-dependent effect. However, there is a need for further investigation into the use of bedside tissue microvascular perfusion parameters as targets for resuscitation in critically ill patients.

## Electronic supplementary material

Below is the link to the electronic supplementary material.

Supplementary material 1 (DOCX 23 kb)

## Data Availability

The datasets analyzed during the current study are available from the corresponding author on reasonable request.

## Abbreviations

BE: Base excess
CI: Cardiac index
CRT: Capillary refill time
CVP: Central venous pressure
HR: Heart rate
ICU: Intensive care unit (ICU)
IQR: Interquartile range
MAP: Mean arterial pressure
NIRS: Near-infrared spectroscopy
PaCO_2_: Partial pressure of arterial carbon dioxide
PaO_2_: Partial pressure of arterial oxygen
PPI: Peripheral perfusion index
SAPS: Simplified acute physiology
SBP: Systolic blood pressure
ScvO_2_: Central venous oxygen saturation
SD: Standard deviation
SOFA: Sequential organ failure assessment
StO_2_: Tissue oxygen saturation
STO_2_max: Maximum tissue oxygen saturation
STO_2_min: Minimal tissue oxygen saturation
THI: Tissue hemoglobin index
Tskin-diff: Skin-temperature gradient
VOT: Vascular occlusion test
