# Supplementary material for: Assessment of the peripheral microcirculation in patients with and without shock: a pilot study on different methods
Source: J Clin Monit Comput. 2019 Nov 21;34(6):1167–76. doi: 10.1007/s10877-019-00423-8 (PMC7548274; doi:10.1007/s10877-019-00423-8)
Supplement: Supplementary file 1 — Supplementary material 1 (DOCX 23 kb) [file 10877_2019_423_MOESM1_ESM.docx]

**ASSESSMENT OF THE PERIPHERAL MICROCIRCULATION IN PATIENTS WITH AND WITHOUT SHOCK: A PILOT STUDY INCLUDING NEAR-INFRARED SPECTROSCOPY**

*Journal of Clinical Monitoring and Computing*

**Supplementary material**

**Authors:**

Roberto Rabello Filho, MD; Renato Carneiro de Freitas Chaves, MD; Murillo Santucci Cesar Assunção, MD MSc; Ary Serpa Neto, MD MSc PhD; Flavia Manfredi De Freitas, MSc; Maria Laura Romagnoli, RN, Eliézer Silva, MD PhD; Bernardo Lattanzio, MD; Arnaldo Dubin, MD PhD; Thiago Domingos Corrêa, MD PhD.

**Corresponding author:**

Roberto Rabello Filho

Hospital Israelita Albert Einstein, Intensive Care Unit.

Av. Albert Einstein, 627/701, 5th floor, São Paulo, Brazil.

Phone: +55 11 21510603

ZIP CODE: 05651-901

E-mail: [roberto.rabello@einstein.br](mailto:thiago.correa@einstein.br)

**Table S1**. Linear regression and correlation between SOFA score and perfusion parameters.

| **Characteristics** | **ß_1_** | **IC95%** | **r** | **R^2^** | **P value** |
| --- | --- | --- | --- | --- | --- |
| CRT | 1.481 | 0.357 – 2.604 | 0.402 | 0.162 | 0.011 |
| Tskin-diff | -0.010 | -0.472 – 0.453 | -0.007 | 0.000 | 0.966 |
| PPI | -0.124 | -0.842 – 0.594 | -0.056 | 0.003 | 0.730 |
| StO_2_, % | -0.073 | -0.209 – 0.062 | -0.175 | 0.030 | 0.281 |
| StO_2_ min, % | -0.082 | -0.170 – 0.006 | -0.293 | 0.086 | 0.067 |
| StO_2_ max, % | -0.130 | -0.282 – 0.021 | -0.271 | 0.074 | 0.090 |
| Descending slope, %/min | 0.058 | -0.239 – 0.355 | 0.066 | 0.004 | 0.695 |
| Ascending slope, %/sec | -0.503 | -1.308 – 0.303 | -0.206 | 0.043 | 0.214 |
| Recovery time, sec | 0.041 | -0.007 – 0.088 | 0.278 | 0.077 | 0.091 |
| StO_2_max - StO_2_min, % | -0.079 | -0.317 – 0.159 | 0.109 | 0.012 | 0.504 |
| Hyperemia area | -0.051 | -0.174 – 0.072 | -0.138 | 0.019 | 0.409 |

CRT: capilary refill time, Tskin-diff: skin-temperature gradient, PPI: peripheral perfusion index, StO_2_: tissue oxygen saturation, StO_2_min: minimum StO_2_ after arterial occlusion test, StO_2_max: maximum StO_2_ after arterial occlusion. ß_1_: regression slope, r: Pearson correlation coefficient, IC95%: 95 confident intervals, R^2^: r squared.
